# Interleukin-27 Early Impacts *Leishmania infantum* Infection in Mice and Correlates with Active Visceral Disease in Humans

**DOI:** 10.3389/fimmu.2016.00478

**Published:** 2016-11-04

**Authors:** Begoña Pérez-Cabezas, Pedro Cecílio, Ana Luisa Robalo, Ricardo Silvestre, Eugenia Carrillo, Javier Moreno, Juan V. San Martín, Rita Vasconcellos, Anabela Cordeiro-da-Silva

**Affiliations:** ^1^i3S – Instituto de Investigação e Inovação em Saúde, Universidade do Porto, Porto, Portugal; ^2^IBMC – Instituto de Biologia Molecular e Celular, Universidade do Porto, Porto, Portugal; ^3^ICVS – Instituto de Investigação em Ciências da Vida e Saúde, Escola de Ciências da Saúde, Universidade do Minho, Braga, Portugal; ^4^ICVS/3B’s – Laboratório Associado, Braga, Portugal; ^5^WHO Collaborating Centre for Leishmaniasis, Centro Nacional de Microbiología, Instituto de Salud Carlos III, Madrid, Spain; ^6^Hospital Universitario de Fuenlabrada, Madrid, Spain; ^7^Departamento de Imunobiologia, Instituto de Biologia, Universidade Federal Fluminense, Niterói, Brazil; ^8^Faculdade de Farmácia, Departamento de Ciências Biológicas, Universidade do Porto, Porto, Portugal

**Keywords:** IL-27, *Leishmania infantum*, human, mouse models, immune regulation

## Abstract

The complexity of *Leishmania*–host interactions, one of the main leishmaniasis issues, is yet to be fully understood. We detected elevated IL-27 plasma levels in European patients with active visceral disease caused by *Leishmania infantum*, which returned to basal levels after successful treatment, suggesting this cytokine as a probable infection mediator. We further addressed this hypothesis recurring to two classical susceptible visceral leishmaniasis mouse models. BALB/c, but not C57BL/6 mice, showed increased IL-27 systemic levels after infection, which was associated with an upregulation of IL-27p28 expression by dendritic cells and higher parasite burdens. Neutralization of IL-27 in acutely infected BALB/c led to decreased parasite burdens and a transient increase in IFN-γ^+^ splenic T cells, while administration of IL-27 to C57BL/6 promoted a local anti-inflammatory cytokine response at the site of infection and increased parasite loads. Overall, we show that, as in humans, BALB/c IL-27 systemic levels are infection dependently upregulated and may favor parasite installation by controlling inflammation.

## Introduction

The 2013 Global Burden of Disease reveals leishmaniasis as one of the deadliest neglected tropical infectious diseases, affecting millions of people worldwide (1). It is caused by the sandfly transmitted protozoan parasite *Leishmania*. The infection can be asymptomatic or manifest in cutaneous, mucosal, and visceral forms, the last one being fatal if left untreated (2). These forms depend mostly on the infecting parasite species but also on the efficiency of the host immune response, especially during the first moments after parasite inoculation (3). Phagocytic cells are rapidly recruited upon *Leishmania* deposition in the skin by the sandfly, but the parasite is able to manipulate them using different strategies. One of the effects of this immunomodulation is the alteration of cytokines secretion in the infection microenvironment. Interferences with receptors and signaling pathways prevent the production and the function of pro-inflammatory cytokines such as IL-12 and IFN-γ and favors anti-inflammatory IL-10 and TGF-β contributing to parasite survival [reviewed in Ref. (4)].

Interleukin-27 is composed of the subunits p28 and EBV-induced protein 3 (EBI3) that interacts with a receptor comprising the glycoprotein 130 (gp130) and IL-27Rα or WSX-1. Interleukin-27 is mainly produced by macrophages and dendritic cells (DCs) after the stimulation of surface receptors such as TLRs or CD40 and following IFNs signaling (5, 6). Although this cytokine was initially described as pro-inflammatory, numerous anti-inflammatory and immunomodulatory properties are recognized today [reviewed in Ref. (5, 6)].

In the past few years, several reports have contributed to the understanding of the IL-27 role during *Leishmania* infection. Active visceral leishmaniasis (VL) patients from India (7) and Brazil (8) present increased IL-27 in plasma. In mice, IL-27 appears to be essential to prevent severe immunopathology after infection with both cutaneous (9) and visceral (10) strains, mainly through the effects of IL-10. However, whether IL-27 directly affects parasite burdens (9, 11) or if IL-10 appears as a consequence of inflammatory (7, 12) or suppressor (13) events is still unclear.

In the present study, we report elevated IL-27 plasma levels in *Leishmania infantum*-infected European patients with active VL, which normalized after successful treatment. Curiously, IL-27 increased early after *L. infantum* infection in the serum of BALB/c, but not in C57BL/6 mice. We took advantage of this dichotomy to understand the mechanisms underlying the effects of IL-27 in visceral *Leishmania* infection. Early blocking of IL-27 in BALB/c mice decreased parasite loads, while IL-27 administration in C57BL/6 augmented parasite burdens. Immunological studies suggested that IL-27 is produced as a consequence of parasite subversion of the host immune response, resulting in a control of inflammation beneficial for parasite installation.

## Materials and Methods

### Ethics Statement

Human sample collection was in accordance with Good Clinical Practice guidelines. The study was approved by the Ethics Committee of the University Hospital of Fuenlabrada (Madrid, Spain). All subjects provided their written informed consent.

Animal experiments were performed in accordance with the IBMC.INEB Animal Ethics Committees and the Portuguese National Authorities for Animal Health guidelines (directive 2010/63/EU). Begoña Pérez-Cabezas and Anabela Cordeiro-da-Silva are accredited for animal research (Portuguese Veterinary Direction, Ministerial Directive 113/2013).

### Human Studies

Diagnosed VL patients were treated with liposomal Amphotericin B (21 mg/kg). Cure was 100% in 3 months after diagnosis. Subjects without previous VL symptomology were included as negative controls. Plasma was recovered from heparinized blood and stored at −20°C for posterior IL-27 determination using MILLIPLEX MAP (Millipore, Germany) and analyzed on a Bio-Plex-200 Luminex (Bio-Rad, CA, USA) (minimum detectable concentration 0.063 ng/mL).

### Parasites and Mice

A clone of virulent *L. infantum* (MHOM/MA/67/ITMAP-263) was maintained by weekly subpassages at 26°C in RPMI 1640 medium supplemented with 10% heat-inactivated fetal bovine serum (FBS), 2 mM l-glutamine, 100 U/mL penicillin, 100 microgram/mL streptomycin, and 20 mM HEPES buffer (all from BioWhittaker, Lonza, Switzerland). Promastigotes from 4 to 10 passages were used in these experiments.

Six- to eight-week-old male BALB/c and C57BL/6 mice (Charles River Laboratories, France) were maintained under specific pathogen-free conditions at the IBMC facilities. Animals were infected i.p. with 1 × 10^8^ stationary promastigotes from 5 culture days.

### Mice Sera Collection

Blood from mice was collected through intracardiac puncture under isoflurane anesthesia. Serum was collected and stored at −80°C for posterior analysis.

### Splenic Dendritic Cells and Macrophages Cell Sorting and Hepatic Kupffer Cell Enrichment Procedure

Spleen and Liver were aseptically collected from 24 h infected mice. Splenocytes were obtained and labeled to distinguish DCs (CD11b^+^/CD11c^high^) and macrophages (CD11b^+^/Ly6G^−^/Ly6C^+^/F4-80^+^), and sorted using a FACSAria and the FACSDiva software (BD Biosciences, NJ, USA) for posterior RNA analysis. Sorted cells purity was >95%.

Enriched Kupffer cells were obtained after hepatic collagenase digestion, followed by a Percoll density gradient centrifugation (Sigma-Aldrich, MO, USA) and a brief adhesion step, as described elsewhere (14). The percentage of Kupffer cells was determined by flow cytometry (F4/80^+^/CD11b^variable^). Cells were resuspended in lysis buffer and frozen for posterior RNA analysis.

### RNA Extraction and Quantitative RT-PCR

Total RNA was extracted (RNeasy Mini Kit, Qiagen, CA, USA), quantified (NanoDrop1000, Thermo Scientific, MA, USA), and reversely transcribed (NZY-First-Strand cDNA synthesis Kit, NZYTech, Portugal). Quantitative RT-PCRs were run on an iQ5 iCycler thermal cycler (Bio-Rad) (15). Results were analyzed (built-in iQ5 v2, Bio-Rad) and normalized using the reference gene GAPDH. The sequences of the primers used were *IL-27p28* Forward TCGATTGCCAGGAGTGAACC, Reverse CGAAGTGGTAGCGAGGAAG; *EBI3* Forward AGCAGCAGCCTCCTAGCCT, Reverse ACGCCTTCCGGAGGGTC; and *GAPDH* Forward CTGGTCCTGAGTGTAGCCCAA, Reverse CATGGCCTTCCGTGTTCCTA.

### *In Vitro* Differentiation and Infection of Bone Marrow-Derived DCs and Macrophages

For bone marrow-derived DCs (BMDCs) differentiation, 6 × 10^6^ bone marrow cells were seeded in 25 mL of complete RPMI supplemented with 50 μM 2-mercaptoethanol (Sigma-Aldrich) and 10% of GM-CSF-containing supernatant from J558 cell conditioned medium (DC medium) (16). Cells were cultured at 37°C and 5% CO_2_ for 3 days, after which the same amount of DC medium was added to each flask. At day 6, half of the culture supernatant was replaced with fresh DC medium. At day 8, cells were thoroughly resuspended, recovered, counted, and, finally, plated in 96 round-bottom culture plates at 1 × 10^5^ cells/well in 200 μL of DC medium. Bone marrow-derived macrophages (BMMØ) were obtained from non-adherent bone marrow cells collected after an initial overnight incubation (37°C and 5% CO_2_) in complete Dulbecco’s modified Eagle’s medium (DMEM) (Lonza). Non-adherent cells were counted and distributed in 96 flat-bottom well culture plates at 1 × 10^5^ cells/well in 100 μL of complete DMEM supplemented with 5% of l-929 cell conditioned medium (LCCM). After 3 days of culture, 100 μL of DMEM + 5% LCCM were added per well. Half of the media volume was renewed at day 6 of culture. BMMØ acquired a definitive differentiation status at day 8.

Stationary promastigotes from 5 culture days (cultured as described in Section “Parasites and Mice”) were added to BMDC or BMMØ at a 10:1 ratio. Non-internalized parasites were washed after 4 h incubation (37°C and 5% CO_2_). Non-infected cells were stimulated with 1 μg/mL LPS (Sigma-Aldrich) as positive control, or received complete medium as negative control. Supernatants were recovered 24 h post-infection for cytokine quantification.

### *In Vivo* IL-27 Modulation

Twenty-four hours after infection, BALB/c mice were i.p. treated with 20 μg of goat anti-mouse IL-27p28 neutralizing antibody (a-IL-27) and C57BL/6 with 1 μg of mouse recombinant IL-27 (rIL-27) (both from R&D Systems), as previously reported (17, 18). As controls, BALB/c and C57BL/6 received 20 μg of goat IgG (isotype control) and PBS (mock), respectively.

### Determination of Parasite Burdens

Spleen and liver were aseptically collected, weighted, and disrupted. Splenic and hepatic parasite burdens were assessed by the limit dilution method. The parasite titer was considered as the last dilution with >1 motile parasite. The number of parasites per gram of organ was calculated, as discussed previously (19). Peritoneal cells were recovered and 2 × 10^5^ cells from each exudate were subjected to cytospin in 200 μL of PBS during 5 min at 1000 rpm using a Shandon Cytospin II (GMI, MN, USA). Cell preparations were fixed with 2% paraformaldehyde (PFA) for 20 min. Afterward, an adapted staining protocol was performed (20) by 2 min immersion in Kaplow modified solution, followed by 45 s immersions in Hemacolor reagent 1 and reagent 2 (Merk Millipore, Germany). Finally, slides were washed with distilled water, air dried, and observed by optical microscopy (100× magnification). For determination of the percentage of infected cells, 200 consecutive cells were differentially counted (infected versus non-infected) in 3 different areas of the same preparation and the mean of the 3 areas was calculated. The number of parasites per infected cells was assessed by counting 100 different infected cells from which the mean was calculated.

### Flow Cytometry

The anti-mouse monoclonal antibodies used to perform this study were all purchased to BioLegend (CA, USA) except if otherwise stated: FITC-labeled anti-IgM (R6-60.2, BD Biosciences, NJ, USA), anti-MHC-II(I-Ad) (AMS-32.1, BD), anti-MHC-II(I-A/I-E) (M5114.15.2), anti-IFN-γ (XMG1.2), and anti-IL-17A (TC11-18H10.1); PE-labeled anti-CD8 (53-6.7, BD), anti-CD11b (M1/70), anti-Siglec-F (E50-2440, BD), anti-F4/80 (BM8), anti-IL-4 (11B11), and anti-IL-6 (MP5-20F3); PerCP-labeled anti-CD11b (M1/70); PerCP-Cy5.5-labeled anti-Ly6C (HK1.4), anti-F4-80 (BM8), and anti-TNFα (MP6-XT22); PE-Cy7-labeled anti-CD3 (HA2) and anti-CD11b (M1/70); APC-Cy7-labeled anti-CD11c (N418); APC-labeled anti-CD19 (6D5), anti-IL-5 (TRFK5), and anti-IL-10 (JES5-16E3); BV510-labeled anti-CD4 (RM4-5); and Pacific Blue™-labeled anti-Ly6G (1A8).

To analyze lymphoid and myeloid cell populations, two panels of antibodies were designed. The lymphoid panel was composed of anti-CD8, -CD3, -CD4, and -CD19. The Myeloid panel comprised anti-CD11b, -CD11c, -Siglec-F, -Ly6C, -Ly6G, and -MHC-II. Surface staining of peritoneal and splenic cells was performed in PBS + 0.5% BSA (20min, 4°C) followed by 15 min fixation using 1% PFA. For intracellular staining, splenocytes were cultured for 2 h with PMA/Ionomycin (50/500 ng/mL) and then for 2 h with Brefeldin A (10 μg/mL). Cells were surface stained and then intracellularly after fixation and permeabilization with 1% saponin (all from Sigma) (21). Samples were acquired in a FACSCanto (BD) and analyzed using the FlowJo software v10 (TreeStar, OR, USA).

An initial gate plotting FSC-A versus SSC-A was performed. Afterward, singlets were selected by plotting FSC-A versus FSC-H and the remaining cell populations were resolved. T lymphoid cell populations were defined as CD3^+^/CD4^+^ and CD3^+^/CD8^+^, while B cells were defined as CD19^+^. Cytokine production by T cells was assessed within CD3^+^/CD4^+^ and CD3^+^/CD8^+^cells. Myeloid cell populations were gated as eosinophils (Siglec-F^+^/SSC-H^int/high^), neutrophils (CD11b^high^/Ly6G^high^/Siglec-F^−^), DCs (CD11c^+^/MHC-II^int/high^), and macrophages (CD11b^+^/CD11c^−^/Ly6G^−^/Siglec-F^−^).

### Determination of Mouse Cytokines by ELISA

Cytokines were quantified, according to the manufacturer’s instructions, using the commercial kits: IL-27 ELISA Ready-SET-Go!^®^ (eBiosciences, CA, USA) (detection limit 16 pg/mL); IL-27p28/IL-30 and IL-10 DuoSet ELISA (R&D Systems, MN, USA) (detection limits 15.6 and 31.2 pg/mL, respectively); IL-12p70 and IFN-γ ELISA MAX Deluxe (BioLegend, CA, USA) (detection limit 4 pg/mL for both cytokines).

### Statistical Analysis

Results are expressed as mean ± SEM. Statistical differences were analyzed using GraphPad Prism v6.01 (CA, USA). Comparisons between human samples were performed using Mann–Whitney test for unpaired samples and Wilcoxon test for paired data. Mice experimental groups were compared using the unpaired *t*-test unless otherwise stated.

## Results

### Systemic IL-27 Increases in *L. infantum*-Infected European Patients and BALB/c Mice

**Figure 1:**
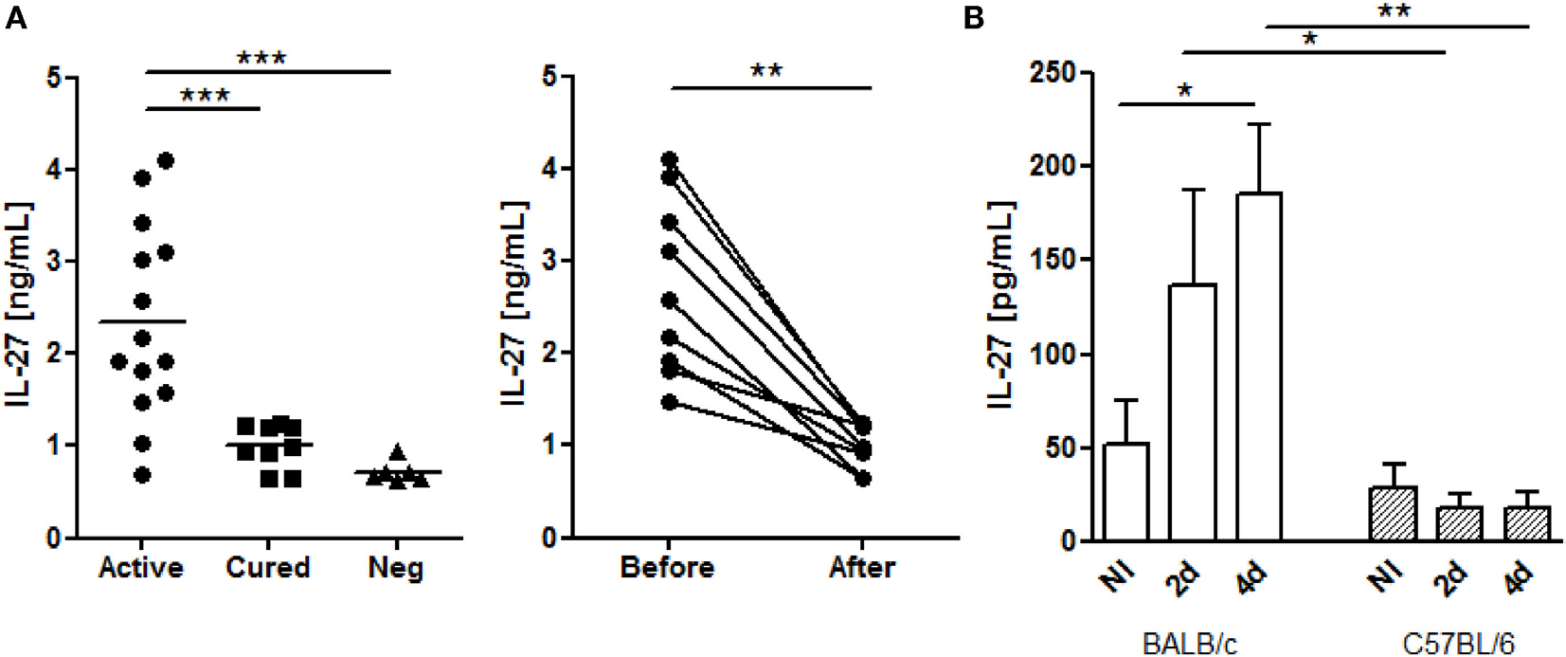
***L. infantum* infection increases systemic IL-27 in European VL patients and in BALB/c mice**. **(A)** Interleukin-27 levels in the plasma of European patients with active VL caused by *L. infantum* (black dots, *n* = 14), cured patients (black squares, *n* = 9), and negative controls (black triangles, *n* = 6). The cytokine was also determined in VL patients before and after treatment (*n* = 9). Each symbol represents one individual; bars represent the mean of the experimental groups. Mann–Whitney test was used to compare clinical groups and Wilcoxon matched pairs test for paired comparisons. **(B)** Interleukin-27 levels in the serum of BALB/c (white columns) and C57BL/6 (patterned columns) mice, infected i.p. with 1 × 10^8^ *L. infantum* promastigotes for 2 and 4 days. Bars represent mean ± SEM of three independent experiments with a minimum of four animals per group and experiment. Unpaired *t*-test was used to assess statistical significances (*p ≤ 0.05, **p ≤ 0.01, and ***p ≤ 0.001).

To address if the increase of systemic IL-27 is a common fact of VL among different endemic areas (7, 8), we determined this cytokine in the plasma of active *L. infantum* infected individuals from a current outbreak in Spain. Patients with active disease presented higher IL-27 levels than both cured (*p* ≤ 0.001) and negative controls (*p* ≤ 0.001) (Figure 1A). Some patients were re-sampled after cure, which confirmed that IL-27 decreases after successful treatment (*p* ≤ 0.01) (Figure 1A).

The role of IL-27 during *Leishmania* infection was addressed using mouse models by several authors (10–13, 22). However, whether IL-27 is also increased in the serum of *Leishmania*-infected mice has never been shown. Therefore, we addressed if BALB/c and C57BL/6, two susceptible VL mouse species, displayed a similar systemic increase of IL-27 as observed in *L. infantum*-infected humans. While IL-27 remained unchanged in the serum of infected C57BL/6 mice, infected BALB/c showed an early increase that was significant 4 days after infection (*p* ≤ 0.05) and always higher than the IL-27 levels of C57BL/6 mice (*p* ≤ 0.05 comparing the day 2 and *p* ≤ 0.01 the day 4 after infection) (Figure 1B).

### Dendritic Cells Are the Main Cellular Source of IL-27 in Infected BALB/c Mice

**Figure 2:**
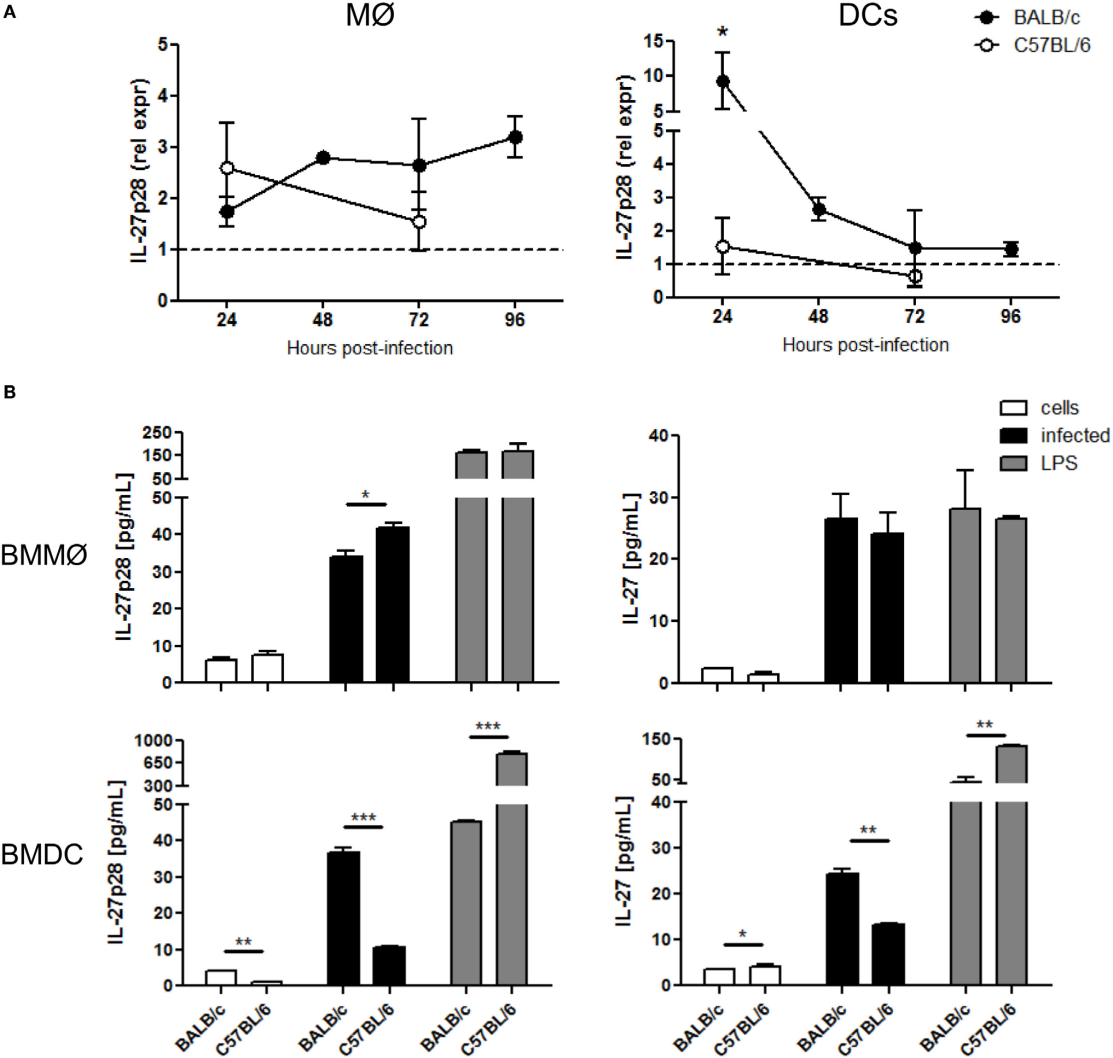
**Dendritic cells are the main source of IL-27 after *L. infantum* infection in BALB/c mice**. **(A)** Splenic MØ and DCs were sorted from infected BALB/c (black symbols) and C57BL/6 (open symbols) mice at the indicated time points. The transcription levels of IL-27p28 were quantified by qRT-PCR. Results are relativized to non-infected mice values (=1). Every symbol and bars represent the mean and SEM, respectively, of four different animals per group analyzed in two different independent experiments. Unpaired *t*-test was used to assess statistical significances. **(B)** The secretion of IL-27p28 and IL-27 was quantified by ELISA in 24 h supernatants of BMMØ and BMDC infected with *L. infantum* promastigotes at a 1:10 ratio (black bars). Medium (white bars) and LPS (gray bars) were used as negative and positive controls, respectively. Bars represent the mean ± SEM of two independent experiments, every run in duplicate. Unpaired *t*-test was used to assess statistical significances (*p ≤ 0.05, **p ≤ 0.01, and ***p ≤ 0.001).

As the main sources of IL-27 are myeloid cell populations, and macrophages and DCs play critical roles during *Leishmania* infection, we decided to discriminate their contribution to IL-27 response after *L. infantum* infection. For that, the expression of IL-27 subunits in splenic DCs and macrophages sorted from infected BALB/c and C57BL/6 was evaluated. While the expression of EBI3 remained always comparable to basal levels (Figure S1 in Supplementary Material), IL-27p28 expression was significantly upregulated but only in DCs from BALB/c 24 h after infection (*p* ≤ 0.05) (Figure 2A). The contribution of Kupffer cells, the resident liver macrophages, for the IL-27 response to *L. infantum* was also addressed. The expression of IL-27 subunits was analyzed in Kupffer cells from non-infected and infected BALB/c mice. However, no increase in the RNA levels of IL-27p28 and EBI3 was detected in these cells 24 h after infection (Figure S2 in Supplementary Material), suggesting that DCs, and not macrophages, are the main cell source responsible for the increase of IL-27 in our model.

To confirm our data at protein level, we determined IL-27 and IL-27p28 in supernatants from 24 h *L. infantum*-infected BMMØ and BMDCs. Although after infection the production of IL-27p28 was greater in BMMØ from C57BL/6 than from BALB/c (*p* ≤ 0.05), IL-27 concentration was similar in both mouse strains. No difference was found after LPS activation (Figure 2B). In line with *ex vivo* results, both IL-27p28 and IL-27 secretion by BMDCs were significantly higher in cells from BALB/c when compared to C57BL/6 (*p* ≤ 0.001 and *p* ≤ 0.01, respectively) (Figure 2B). Interestingly, the ability of BMDCs to produce both cytokines after LPS stimulation was higher in C57BL/6 than in BALB/c, indicating that these cells are really capable of producing IL-27, but not in response to *L. infantum* infection. These results suggest that the parasite can actively modulate the secretion of IL-27 and IL-27p28 in BMDCs obtained from BALB/c, but not from C57BL/6 mice.

### IL-27 Favors *L. infantum* Infection in Mouse Models

**Figure 3:**
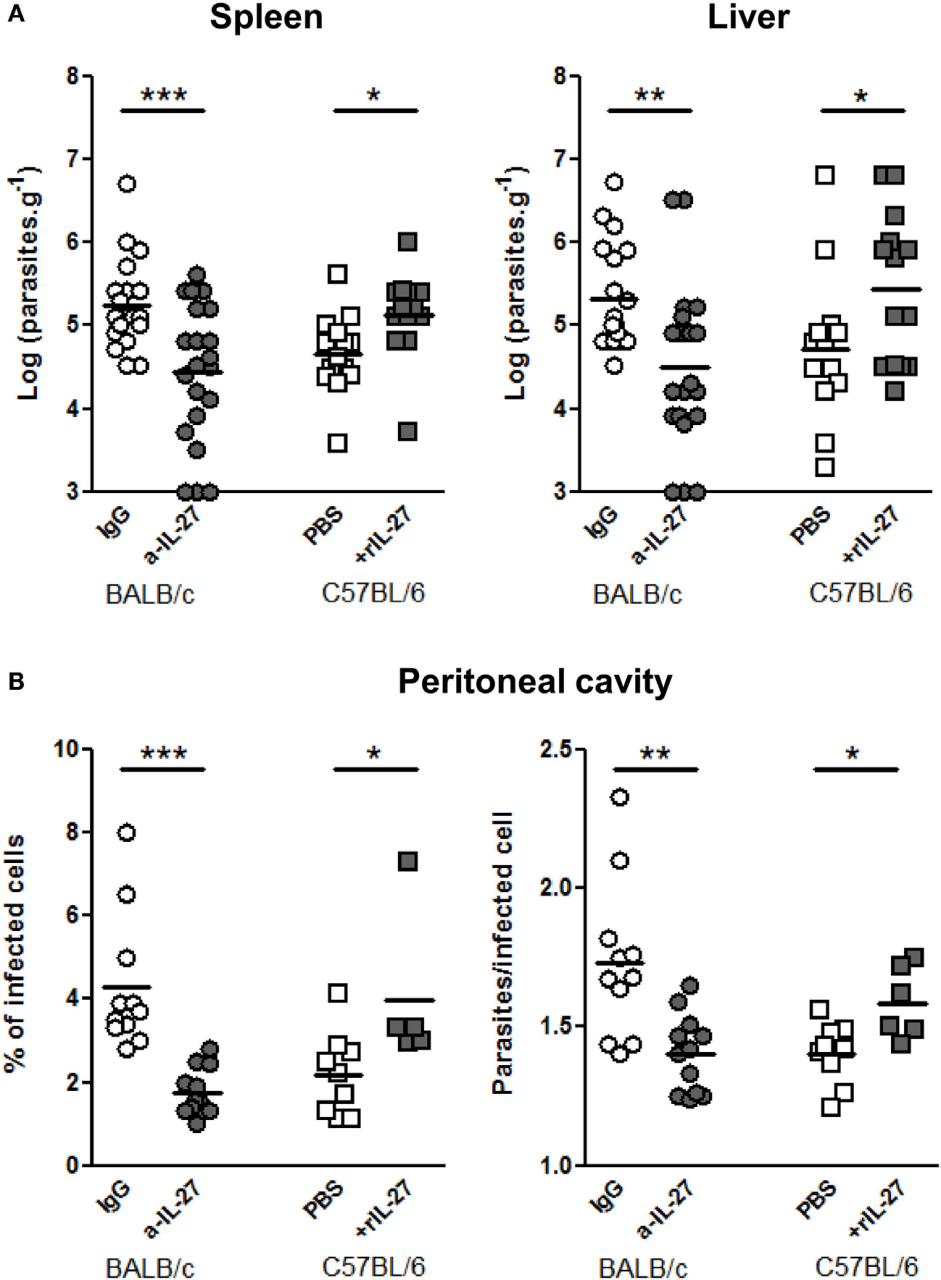
**IL-27 favors *L. infantum* infection in mice models**. **(A)** BALB/c (circles) and C57BL/6 (squares) mice were i.p. infected with 1 × 10^8^ promastigotes. Twenty-four hours later, BALB/c mice were treated i.p. with 20 μg of a-IL-27 (gray circles) or IgG isotype control (open circles), while C57BL/6 received i.p. 1 μg of mouse rIL-27 (gray squares) or the same volume of PBS (open squares). Three days after treatment, mice were euthanized and the parasite load of spleen and liver determined by limiting dilution. **(B)** In some animals, the peritoneal cavity was washed for collection of recruited cells. Approximately 2 × 10^5^ cells from every exudate were cytospined, fixed, and stained with Kaplow modified solution and by the Giemsa method. Finally, slides were observed by optical microscopy at 100× for determination of the percentage of infected cells and the number of parasites/infected cell. All data represent results obtained in three independent experiments. Every symbol represents a mouse and the bars the mean of the group. Unpaired *t*-test was used to assess statistical significances (*p ≤ 0.05, **p ≤ 0.01, and ***p ≤ 0.001).

To determine whether IL-27 contribute to the infection outcome, we neutralized IL-27 in BALB/c and administered rIL-27 to C57BL/6 mice 24 h after infection, coinciding with the IL-27p28 RNA peak in the spleen and before the IL-27 increase observed in the serum, 2 days after the infection, in BALB/c mice. Seventy-two hours later, these treatments significantly decreased in BALB/c and increased in C57BL/6 splenic and hepatic parasite burdens (Figure 3A). We also observed that IL-27 significantly affected the percentage of infected cells and the number of parasites per infected cells in the peritoneal cavity (Figure 3B). These results revealed a relation between the levels of IL-27 and the establishment of the infection.

### IL-27 Modulates the Cytokine Response in Infected Mice

**Figure 4:**
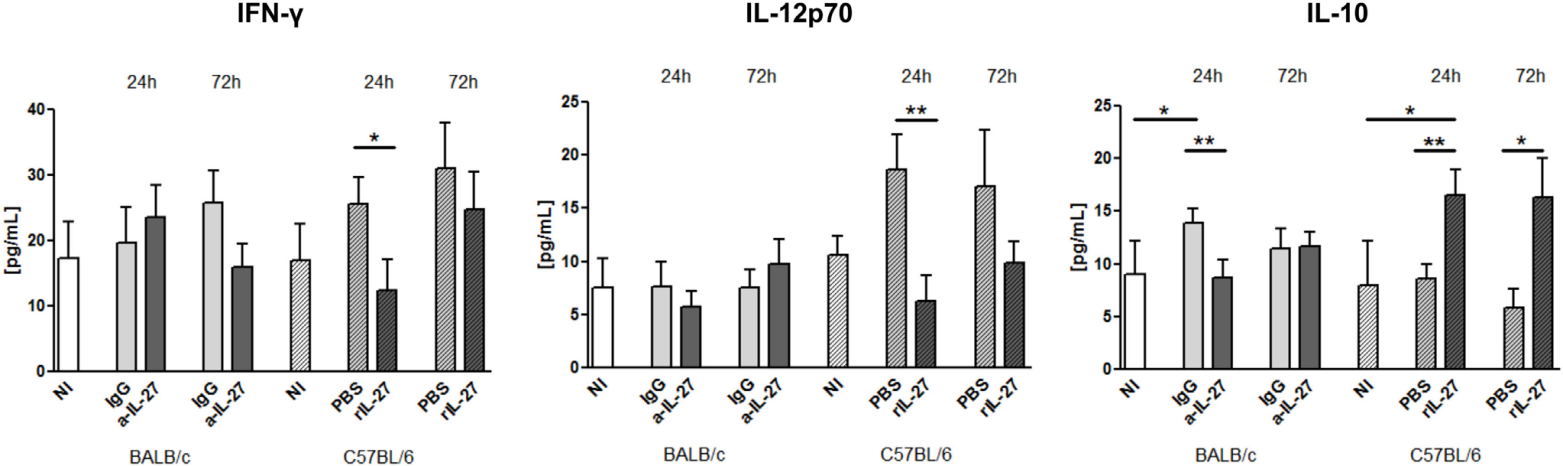
**IL-27 influences the cytokine responses in *L. infantum*-infected mice at the infection site**. BALB/c and C57BL/6 mice were i.p. infected with 1 × 10^8^ promastigotes. Twenty-four hours later, BALB/c mice were treated i.p. with 20 μg of a-IL-27 (clear dark-gray bars) or IgG isotype control (clear light-gray bars), while C57BL/6 received i.p. 1 μg of mouse rIL-27 (patterned dark-gray bars) or the same volume of PBS (patterned light-gray bars). Twenty-four or 72 h after treatment, mice were euthanized and the peritoneal cavity washed. Non-infected (NI) counterparts were always used as controls (white bars, non-patterned for BALB/c and patterned for C57BL/6 mice). The concentration of IFN-γ, IL-12p70, and IL-10 in the peritoneal exudates was determined by ELISA. Bars represent the mean ± SEM of three independent experiments, a minimum of four animals per condition and experiment was analyzed. Unpaired *t*-test was used to assess statistical significances (*p ≤ 0.05 and **p ≤ 0.01).

**Figure 5:**
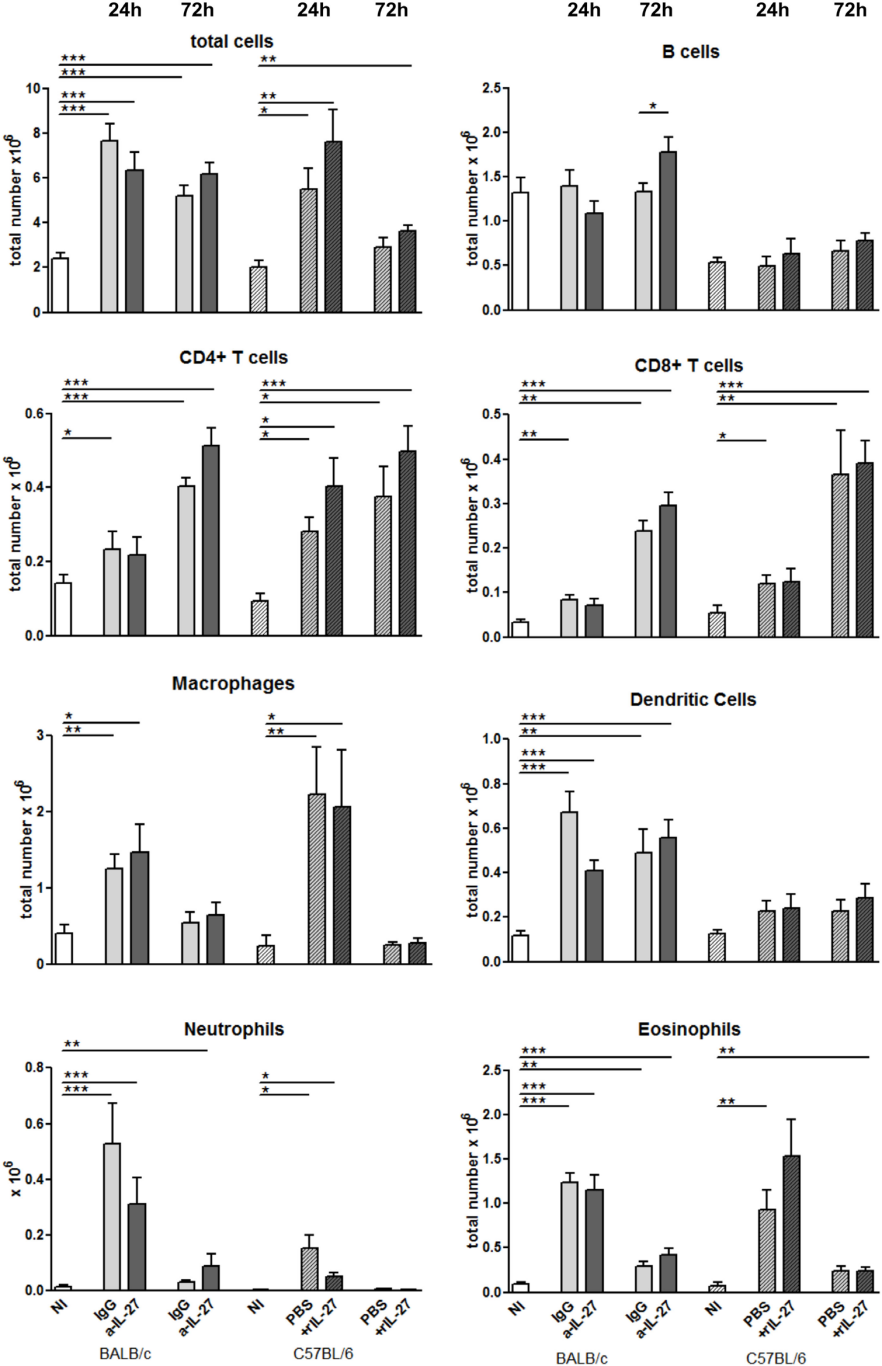
**Cell recruitment to the peritoneal cavity in response to *L. infantum* infection after IL-27 modulation**. BALB/c and C57BL/6 mice were i.p. infected with 1 × 10^8^ promastigotes. Twenty-four hours later, BALB/c mice were treated i.p. with 20 μg of a-IL-27 (clear dark-gray bars) or IgG isotype control (clear light-gray bars), while C57BL/6 received i.p. 1 μg of mouse rIL-27 (patterned dark-gray bars) or the same volume of PBS (patterned light-gray bars). Twenty-four or 72 h after treatment, mice were euthanized and the peritoneal cavity washed. Non-infected (NI) counterparts were always used as controls (white bars, non-patterned for BALB/c and patterned for C57BL/6 mice). Peritoneal cells were then extracellularly stained and acquired by flow cytometry. Bars represent the mean ± SEM of three independent experiments, a minimum of four animals per condition and experiment was analyzed. Unpaired *t*-test was always used to assess statistical significances (*p ≤ 0.05, **p ≤ 0.01, and ***p ≤ 0.001).

**Figure 6:**
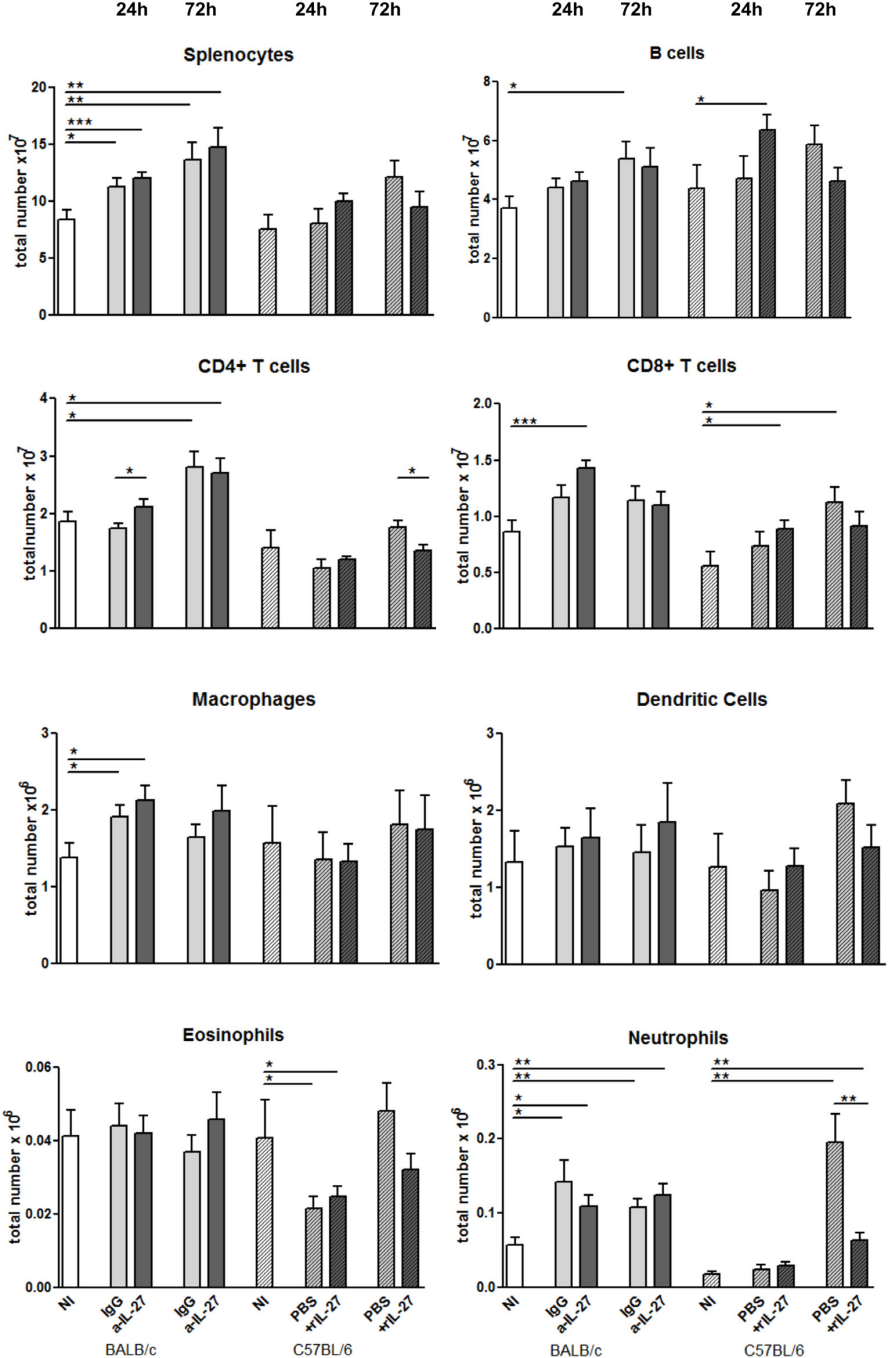
**Cellular composition of the spleen from *L. infantum*-infected mice after IL-27 modulation**. BALB/c (clear bars) and C57BL/6 (patterned bars) mice were infected i.p. with 1 × 10^8^ promastigotes. Twenty-four hours after infection, BALB/c mice were treated i.p. with 20 μg of IL-27 neutralizing antibody (a-IL-27, dark-gray bars) or IgG isotype control (IgG, light-gray bars), while C57BL/6 received i.p. 1 μg of mouse recombinant IL-27 (+rIL-27, dark-gray patterned bars) or the same volume of PBS (PBS, light-gray patterned bars). Non-infected (NI) counterparts were always used as controls (white bars, clear for BALB/c and patterned for C57BL/6 mice). Twenty-four or 72 h after treatment, mice were euthanized and the spleen collected and homogenized. Splenocytes were counted using an automatic cell counter, washed, and phenotyped by flow cytometry. Bars represent the mean and SEM of the three independent experiments, a minimum of four animals was analyzed per condition and experiment. Unpaired *t-*test was used to assess statistical significances (*p ≤ 0.05, **p ≤ 0.01, and ***p ≤ 0.001).

**Figure 7:**
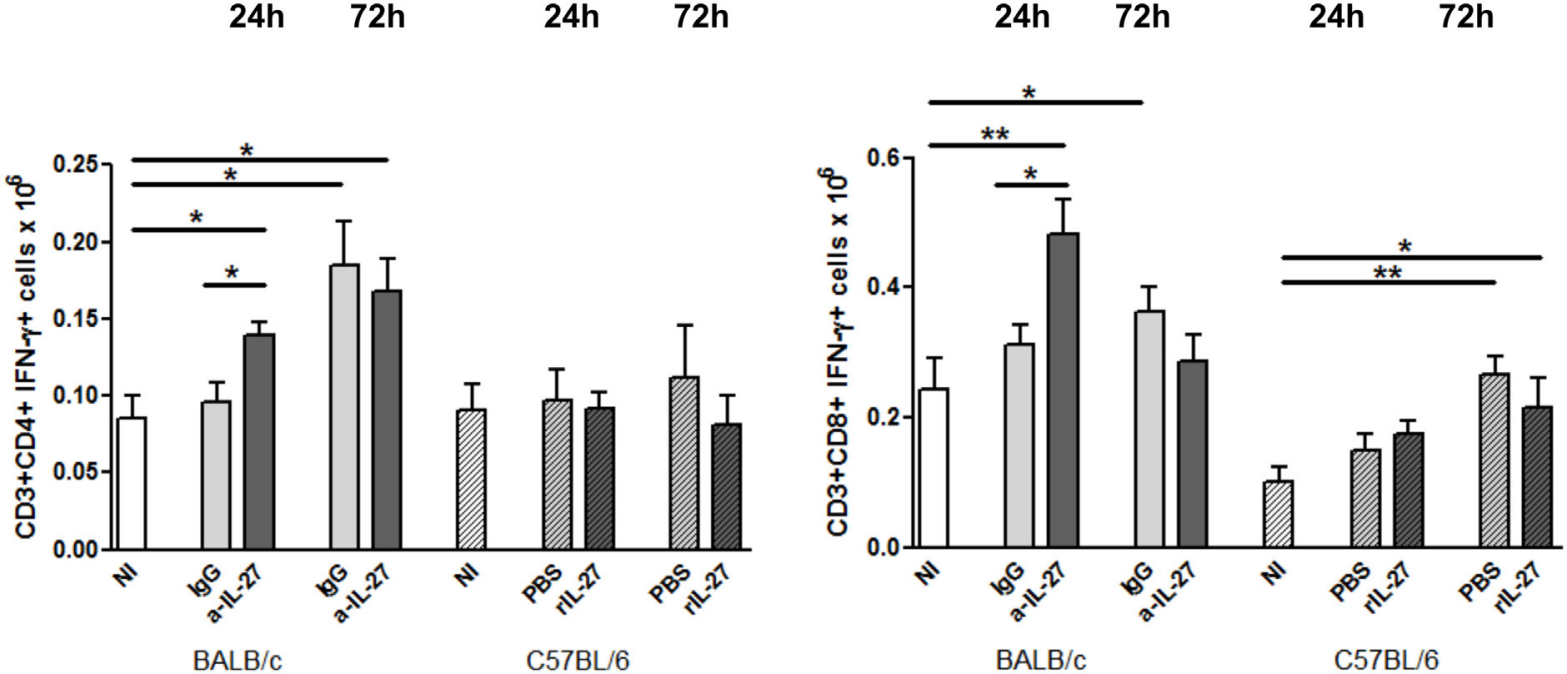
**IL-27 neutralization increases the IFN-γ response of splenic T cells in BALB/c mice (clear bars) and C57BL/6 (patterned bars) mice were infected i.p. with 1 × 10^8^ promastigotes**. Twenty-four hours after infection, BALB/c mice were treated i.p. with 20 μg of IL-27 neutralizing antibody (a-IL-27, dark-gray bars) or IgG isotype control (IgG, light-gray bars), while C57BL/6 received i.p. 1 μg of mouse recombinant IL-27 (+rIL-27, dark-gray patterned bars) or the same volume of PBS (PBS, light-gray patterned bars). Non-infected (NI) counterparts were always used as controls (white bars, clear for BALB/c and patterned for C57BL/6 mice). Twenty-four or 72 h after treatment, mice were euthanized and the spleen collected and homogenized. Splenocytes were counted with an automatic cell counter, washed, and *in vitro* cultured during 4 h in the presence of PMA + Ionomycin and Brefeldin A. Cells were then extra- and intracellularly stained and acquired by flow cytometry. Bars represent the mean and SEM of the three independent experiments, a minimum of four animals was analyzed per condition and experiment. Unpaired *t*-test was used to assess statistical significances (*p ≤ 0.05 and **p ≤ 0.01).

To elucidate how IL-27 interferes at the early stages of *L. infantum* infection, we performed a kinetic study of the peritoneal and splenic compartments after IL-27 modulation. Interestingly, IL-27 neutralization in infected BALB/c mice transiently decreased IL-10 in the peritoneal cavity (*p* ≤ 0.01), and the administration of rIL-27 increased the presence of this cytokine in infected C57BL/6 mice 24 h (*p* ≤ 0.01) and 72 h (*p* ≤ 0.05) after treatment, always comparing with control infected animals (Figure 4). In addition, rIL-27 in C57BL/6 promoted a reduction of IFN-γ (*p* ≤ 0.05) and IL-12p70 (*p* ≤ 0.01) 24 h after treatment in comparison with infected non-treated animals (Figure 4). However, this shift in the cytokine profile determined by IL-27 almost did not alter cell recruitment to the site of infection, as when comparing treated and non-treated animals only the presence of B cells in the peritoneal cavity significantly increased 72 h after IL-27 blockage in BALB/c mice (Figure 5). In the spleen, the number of CD4^+^ T cells increased in BALB/c mice 24 h after IL-27 blockage (*p* ≤ 0.05), and the supply of the cytokine decreased the presence of the same cells in C57BL/6 mice after 72 h (*p* ≤ 0.05) (Figure 5). The administration of rIL-27 also prevented the infiltration of neutrophils in the spleen of C57BL/6 mice (*p* ≤ 0.01) (Figure 6). Analysis of the cytokine production revealed that IL-27 neutralization increased the numbers of IFN-γ producing CD4^+^ and CD8^+^ T cells 24 h post-treatment (Figure 7) (*p* ≤ 0.01). These differences were not detected at 72 h, likely indicating a reversible effect (Figure 7). The potential secretion of other cytokines was also analyzed but no differences between α-IL-27 and IgG receiving mice were observed (Figure S3 in Supplementary Material). In contrast, rIL-27 did not alter the cytokine response of C57BL/6 splenic T cells (Figure 7; Figure S3 in Supplementary Material).

## Discussion

Interleukin-27 is a cytokine with known immunomodulatory properties involved in the pathogenesis of numerous diseases (5, 6). Several types of infections also course with an increase of IL-27 (23–25). However, whether this fact is a simple host mechanism to control inflammation or a complex pathogen evasion strategy is still unknown. *Leishmania* spp. are expert in modulating immune activity through diverse strategies (4). Previous reports on human leishmaniasis indicate that IL-27 increases when the disease is active in both cutaneous (26–28) and visceral forms (7, 8). Here, for the first time, plasma levels of IL-27 were evaluated in *L. infantum*-infected individuals from Europe. This cytokine was significantly increased in the active phase of our VL patients returning to homeostatic levels after treatment. Interestingly, a relapsing case presented increased circulating IL-27, and positive IL-27 results were obtained even when immunofluorescence antibody test (IFAT) was doubtful (data not shown). Further analysis using a larger cohort of patients will help to better understand the value of IL-27 as a complementary biomarker for human VL diagnosis and even for treatment efficacy monitoring, as has been postulated for this disease (8) and other infections (29).

The role of IL-27 during *Leishmania* infection has been addressed using animal models by several groups (9, 10, 12, 30). The general conclusion was that IL-27 controls inflammation and pathology through limitation of IFN-γ (12) and IL-17 (9) production by CD4^+^ T cells, resulting in a permissive environment for the infection. However, these results are based on experiments performed on WSX-1^−/−^ or EBI3^−/−^ mice, presenting two main limitations. First, both WSX-1 and EBI3 are subunits shared with the regulatory cytokine IL-35 (31), meaning that the over-inflammation observed in these knockout mice can be a result of the additive loss of the regulatory function of both cytokines. The second issue is that both mice models were generated in the C57BL/6 genetic background. Although both C57BL/6 and BALB/c strains are considered susceptible models for experimental VL (32), here we showed that only BALB/c and not C57BL/6 mice increased IL-27 in the serum early after i.p. infection with *L. infantum*. The difference observed in the IL-27 production between the two mice strains in this study was confirmed at the cellular level, as splenic DCs from BALB/c but not from C57BL/6 mice upregulated the expression of IL-27p28 after infection. Upregulation of IL-27p28 in splenic DCs from BALB/c mice was previously shown after i.v. infection with *Leishmania donovani*, being CD8α^+^ DCs the major responsible subset (22). The production of IL-27 could restrict the typical Th1 polarization induced by these APCs, known to be essential for parasite limitation (33). In fact, in a conditional IL-27p28 knockout mice model restricted to DCs, CD4^+^ T cell IFN-γ response was exacerbated (34). Our data suggest that DCs are the main cell source responsible for the increase of IL-27 in BALB/c mice early after *L. infantum* infection probably as a result of active cell function modulation by the parasite. One explanation for the IL-27 divergence observed between the mice strains could be a differential expression of molecules involved in the recognition of parasite structures. An example is TLR-2, more expressed by BALB/c than by C57BL/6 mice (35). It has been demonstrated that TLR-2 signaling induces IL-27 production by respiratory epithelial cells (36). Therefore, this natural high expression of TLR-2 by BALB/c may favor the IL-27 increase early after infection, lowering inflammation, and promoting infection.

We exploited the IL-27 dichotomy in BALB/c and C57BL/6 mice to study the role of this cytokine during the early steps of infection, using an *in vivo* artificial-modulation approach. The resulting data revealed a relation between the levels of IL-27 and infection establishment, as the neutralization or the supply of IL-27 resulted in decreased or increased parasite burdens, respectively. In addition, the administration of rIL-27 significantly increased the production of IL-10 while decreased IFN-γ and IL-12p70 in the peritoneal cavity of C57BL/6 mice. Furthermore, the analysis of the cytokine production by splenic T cells revealed that IL-27 neutralization in BALB/c temporarily increased the numbers of IFN-γ producing CD4^+^ and CD8^+^ T cells. These results partially explain the higher parasite burdens quantified in the presence of IL-27. Addition of IL-27 *in vitro* exacerbates the infection of human macrophages by *Leishmania amazonensis via* IL-10 (11) and combined production of IL-27 and IL-10 by *L. donovani*-infected DCs is essential for IL-10 production by Th1 cells, resulting in parasite persistence (13). A recent work in EBI3^−/−^ mice suggested a role for IL-27 in controlling IL-17 production and neutrophil infiltration during the chronic phase of *L. infantum* infection (30). However, whether the absence of the EBI3 subunit has an impact early after infection was not addressed in this work. We can also suggest that the IL-27 produced in response to infection shall not be a product of inflammation as BALB/c mice present higher parasite loads than C57BL/6 during the first days of infection, a difference that is counteracted by the treatments. Probably, the initial immune response of BALB/c is less inflammatory and less capable of limiting infection installation, suggesting again that the parasites directly induce the production of IL-27 for their own benefit. In fact, the absence of IL-27 can also prevent mycobacterial-induced phagosomal maturation arrest, favoring the elimination of the intracellular pathogen by macrophages depending on IFN-γ (37). In addition, IL-27 can impair the protective immunity to *Mycobacterium tuberculosis* in mice, as WSX-1 deficient T cells accumulate more efficiently in the lesions, showing improved capacity to produce IL-2 and reduced expression of cell death markers (25), functions that may also be altered during *Leishmania* infection.

Altogether, our data demonstrate that IL-27 increases after *L. infantum* infection both in humans and in animal models. These results confirm the involvement of IL-27 in mice models of *Leishmania* visceral pathogenesis, limiting inflammation during the initial stages of the infection and favoring parasite persistence, suggesting that the presence of IL-27 early after infection could influence the host immune response and the progression of the disease. Finally, we would like to highlight that IL-27 has the potential to be a useful biomarker for active human VL and for treatment efficacy monitoring, independently of the etiological agent and the geographical region affected.

## Author Contributions

BP-C, RS, and AC-d-S conceived and designed the experiments; BP-C, PC, AR, and EC performed the experiments; BP-C, PC, RS, EC, JM, JVM, RV, and AC-d-S analyzed the data; JM and AC-d-S contributed with reagents/materials/analysis tools; and BP-C, PC, RV, and AC-d-S wrote the paper.

## Conflict of Interest Statement

The authors declare that the research was conducted in the absence of any commercial or financial relationships that could be construed as a potential conflict of interest. The reviewer CL and handling editor declared their shared affiliation, and the handling editor states that the process nevertheless met the standards of a fair and objective review.
