# Supplementary material for: Interleukin-27 Early Impacts Leishmania infantum Infection in Mice and Correlates with Active Visceral Disease in Humans
Source: Front Immunol. 2016 Nov 4;7:478. doi: 10.3389/fimmu.2016.00478 (PMC5095612; doi:10.3389/fimmu.2016.00478)
Supplement: Supplementary file 1 [file Image_1.PDF]

Supplementary Figure 1

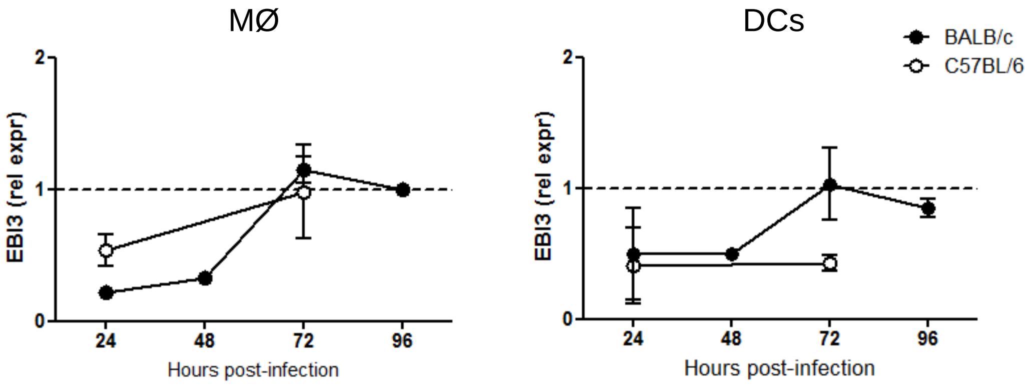

Supplementary Figure 1. *In vivo L. infantum* infection does not alter the expression of EBI3 in splenic macrophages and DCs. BALB/c (black symbols) and C57BL/6 (open symbols) mice were i.p infected with $1x10^8$ promastigotes. Splenic MØ and DCs populations were sorted at the indicated time points post-infection. The transcription levels of EBI3 were quantified by qRT-PCR. Results are expressed relativized to the value obtained from non-infected mice (=1). Every symbol and bars represent the mean and SEM of 4 different animals analyzed in 2 independent experiments.

Supplementary Figure 2

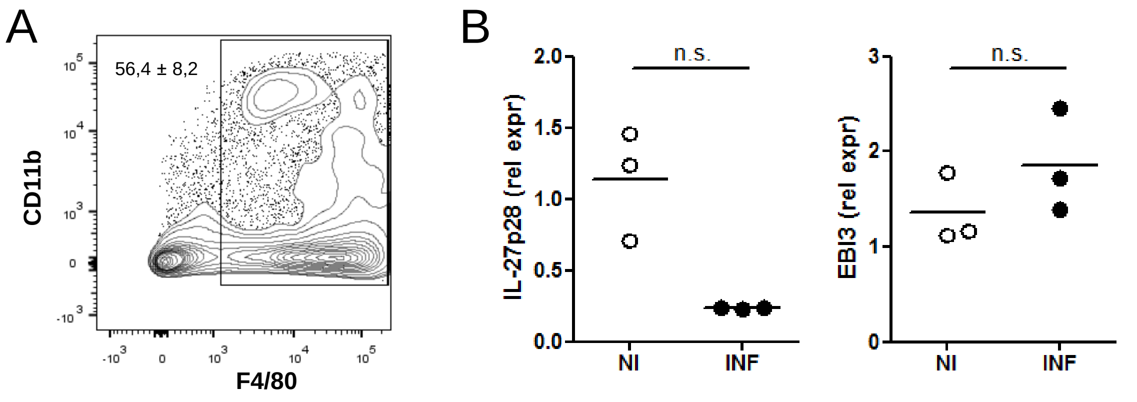

Supplementary Figure 2. Kupffer cells from *L. infantum* infected BALB/c mice do not increase the expression of IL-27p28 and EBI3. BALB/c mice were i.p infected with $1x10^8$ promastigotes or received PBS as control. Twenty-four hours later, kupffer cells were enriched from perfused livers of non-infected and infected mice through a percoll gradient followed by an adhesion step. **(A)** The enrichment of kupffer cells was confirmed by flow cytometry by the expression of F4/80 and CD11b. A representative contour plot is shown. Numbers indicate the mean ± SD of the % of F4/80+ cells in the population obtained after the percoll gradient (n=6, 3 non-infected and 3 infected). **(B)** The transcription levels of IL-27p28 and EBI3 were quantified by qRT-PCR in non-infected (NI, white symbols) and infected mice (INF, black symbols) 24h after the challenge. Every symbol represents a mice and bars the mean of the 3 animals. Mann-Whitney test was used to assess statistical significances, * $p \leq 0.05$.

Supplementary Figure 3

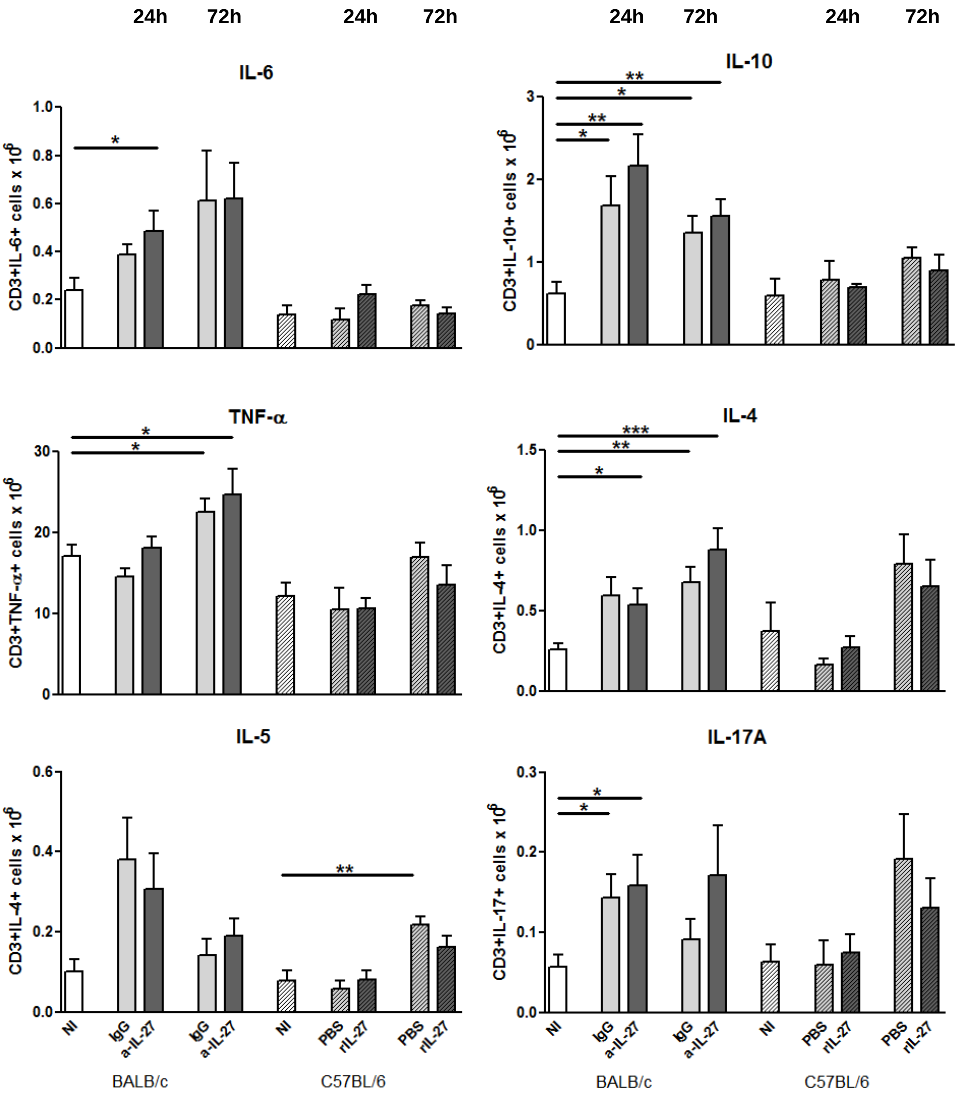

Supplementary Figure 3. Cytokine production by splenic T cells in *L. infantum* infected mice submitted to IL-27 modulation. BALB/c (clear bars) and C57BL/6 (patterned bars) mice were infected i.p. with $1x10^8$ promastigotes. Twenty-four hours after infection, BALB/c mice were treated i.p. with 20μg of IL-27 neutralizing antibody (a-IL-27, dark-grey bars) or IgG isotype control (IgG, light-grey bars), while C57BL/6 received i.p. 1μg of mouse recombinant IL-27 (+rIL-27, dark grey patterned bars) or the same volume of PBS (PBS, light-grey patterned bars). Non-infected (NI) counterparts were always used as controls (white bars, clear for BALB/c and patterned for C57BL/6 mice). Twenty-four or 72 hours after treatment, mice were euthanized and the spleen collected and homogenized. Splenocytes were counted with and automatic cell counter, washed, and in vitro cultured during 4 hours in the presence of PMA+Ionomycin and Brefeldin A. Cells were then extra- and intracellularly stained and acquired by flow cytometry. Bars represent the mean and SEM of the 3 independent experiments, a minimum of 4 animals were analyzed per condition and experiment. Unpaired t test was used to assess statistical significances, * $p \leq 0.05$, ** $p \leq 0.01$, *** $p \leq 0.001$
